# A Spatiotemporal Convolutional Network for Multi-Behavior Recognition of Pigs

**DOI:** 10.3390/s20082381

**Published:** 2020-04-22

**Authors:** Dan Li, Kaifeng Zhang, Zhenbo Li, Yifei Chen

**Affiliations:** College of Information and Electrical Engineering, China Agricultural University, Beijing 100083, China; oliviald@126.com (D.L.); s20183081343@cau.edu.cn (K.Z.); lizb@cau.edu.cn (Z.L.)

**Keywords:** pig, deep learning, spatiotemporal convolutional network, behavior recognition, pig video dataset

## Abstract

The statistical data of different kinds of behaviors of pigs can reflect their health status. However, the traditional behavior statistics of pigs were obtained and then recorded from the videos through human eyes. In order to reduce labor and time consumption, this paper proposed a pig behavior recognition network with a spatiotemporal convolutional network based on the SlowFast network architecture for behavior classification of five categories. Firstly, a pig behavior recognition video dataset (PBVD-5) was built by cutting short clips from 3-month non-stop shooting videos, which was composed of five categories of pig’s behavior: feeding, lying, motoring, scratching and mounting. Subsequently, a SlowFast network based spatiotemporal convolutional network for the pig’s multi-behavior recognition (PMB-SCN) was proposed. The results of the networks with variant architectures of the PMB-SCN were implemented and the optimal architecture was compared with the state-of-the-art single stream 3D convolutional network in our dataset. Our 3D pig behavior recognition network showed a top-1 accuracy of 97.63% and a views accuracy of 96.35% on the test set of PBVD and a top-1 accuracy of 91.87% and a views accuracy of 84.47% on a new test set collected from a completely different pigsty. The experimental results showed that this network provided remarkable ability of generalization and possibility for the subsequent pig detection and behavior recognition simultaneously.

## 1. Introduction

The subclinical or clinical signs of many kinds of pig diseases are often accompanied by behavior changes. Using scientific and effective behavior monitoring methods can increase breeding profits and improve animal welfare. The traditional manual and electronic sensors for monitoring pig behavior has been replaced by computer vision based methods due to the latter’s rapid development and characteristics of automation, contactless and environmental protection.

Currently, various vision based algorithms have been developed to recognize various behaviors of pig. For analyzing lying behavior, ellipse fitting and Delaunay triangulation feature were utilized in the pig group [1]. The modified angle histogram was established according to the optical flow direction for detecting motoring behavior [2]. The faster regions-convolutional neural network (Faster R-CNN) was applied for detecting pigs in the feeding area to observe feeding behavior [3]. The pixel distance of the pig head in drinking water area and the water pipe was calculated for recognizing drinking behavior [4]. Mask region-convolutional neural networks (Mask R-CNN) and kernel-extreme learning were used for detecting mounting behavior [5]. In addition to algorithms for detecting single behavior, there are algorithms for classifying multiple behaviors as well.

Posture classification was used to achieve the purpose of behavior recognition in most of the multiple behavior classification algorithms. By classifying five postures of pigs, Wu implemented recognition of feeding and drinking behaviors [6]. Lying, feeding and drinking behaviors were detected by posture classification in deep images [7]. Faster regions-convolutional neural network (Faster R-CNN), single shot multibox detector (SSD) and region-based fully convolutional network (R-FCN) were applied for classification of lying and other kinds of standing behaviors [8]. Moreover, the behaviors could also be classified by spatiotemporal features. Spatiotemporal interest point and word bag model were applied to represent features of pig’s behavior and support vector machine (SVM) were utilized for classification of feeding, exploring, walking and gathering behavior [9]. A sow behavior detection algorithm based on deep learning (SBDA-DL) was proposed based on MobileNet and SSD for classifying drinking, urination and mounting behavior [10]. All the methods above obtained behavioral information from regions of interest through images. The recognition of behavior depended on the change of pig position. However, for some pig body location invariant behavior, the details cannot be extracted. Therefore, we propose a method based on the characteristics of behavior itself, which uses video to recognize behavior.

Compared with image, video contains the information of temporal sequence. For the task of pig behavior recognition, the processing of temporal series is vital. However, there is no standard video dataset for pig behavior recognition at present, so a pig behavior recognition video dataset (PBVD-5) was built containing five types of behavior, including feeding, lying, motoring, scratching and mounting. Moreover, in order to transfer the fruitful achievements of deep learning to the field of agriculture, we transfer a three-dimensional convolutional neural network, SlowFast network, which is used in human action classification, to pig behavior recognition. Therefore, this paper also proposes a spatiotemporal convolutional network for the pig’s multi-behavior recognition (PMB-SCN) modified from SlowFast. Additionally, the PMB-SCN is compared with the state-of-the-art video action recognition networks and the results show that PMB-SCN provides better performance in the video recognition of pig behavior.

To summarize, our contributions are as follows:

1) A pig behavior recognition video dataset is built including five categories as feeding, lying, motoring, scratching and mounting (PBVD-5).
2) A novel spatiotemporal convolutional network based on SlowFast is proposed for pig’s multi-behavior recognition (PMB-SCN). Variant architectures of the PMB-SCN are implemented and evaluated to explore the optimal architecture.
3) The proposed network is compared with the state-of-the-art video action recognition networks on two test sets, one of which is the test set of the PBVD-5, the other is the new test set collected from a new pigsty. The experimental results show that PMB-SCN provides high degree of accuracy and ability of generalization to some extent, which would be very helpful for the subsequent real-time pig detection and behavior recognition simultaneously.

The rest sections of this paper are arranged as follows. In Section 2, the detailed information of PBVD-5 is provided and in Section 3, the related work of spatiotemporal convolutional networks is introduced and the specific architecture of PMB-SCN is presented. While in Section 4, the material and scheme of the experiment are proposed. In Section 5, the performance of heterogeneous PMB-SCN and the results of the comparison with state-of-the-art spatiotemporal convolutional networks are displayed. Eventually, the discussion and some future research are proposed in Section 6.

## 2. Pig Behavior Video Dataset

### 2.1. Video Acquisition

The videos of pigs were captured 24 hours a day for eighty days from March 23 to June 13, 2018 at China Experimental Miniature Pig Base in Zhuozhou, China. One camera was placed 1 m above the pigsty fence in order to take videos of the daily behaviors of pigs. The size of each pigsty was about 2 m in both length and width. The pig behavior video dataset (PBVD-5) was collected from three pigsties. Three fragrance pigs were placed in each pigsty (due to the death of pig 3, pig 6 and pig 8, in the later period, there were two pigs in each pigsty). The detailed information of pigs was presented in Table 1.

**Figure 1:**
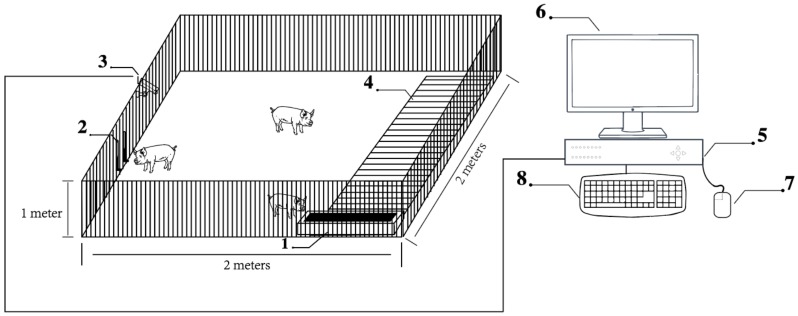
The schematic of video acquisition equipment: 1 feeding trough; 2 drinking nipple; 3 Camera; 4 floor drain; 5 digital video recorder (DVR); 6 monitor; 7 mouse and 8 keyboard.

The camera used in this experiment was Sony infrared camera, and the specification was SSC-CB565R. The host utilized for video recording was the Hikvision digital video recorder (DVR), and the specification was DS-8104HF-ST. The output resolution was 1280×1024/60 Hz, with the framerate set to 25 (PAL)/30(NTSC). The schematic diagram of the video acquisition equipment is shown in Figure 1.

### 2.2. Multi-Behavior Categories of PBVD-5

The healthy physiological state of the pig could be observed through their daily behaviors. In this paper, five kinds of behaviors, feeding, lying, motoring, scratching and mounting were extracted. Among them, daily feeding, lying and motoring behavior could reflect whether pigs were in the normal state of health in the current period. For example, if a pig is suffering from disease, its intake and activity volume will reduce, meanwhile its lying time will increase, etc. Scratching is a kind of grooming behaviors, which is manifested as friction and shaking. The purpose of grooming behavior is to reduce or eliminate external stimulation such as external parasites, mosquitoes, flies and scales, and to smooth and comb hair. If the grooming behavior of a pig reduces, it indicates that the health condition of this pig is abnormal. Mounting behavior can reflect the sexual maturity of pigs, and the pigs should be isolated to avoid skin injury and fracture while this behavior occurs. The description of these behaviors is shown in Table 2.

### 2.3. PBVD-5 Production

**Figure 2:**
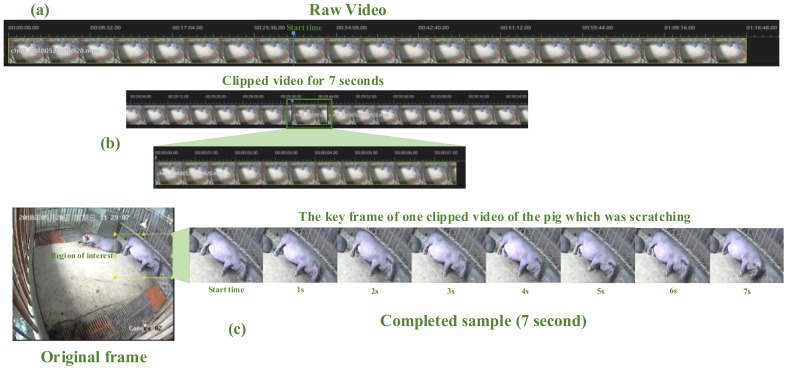
The process of clipping videos (Taking 7-second clip as an example): (**a**) raw video; (**b**) clipped video sample and (**c**) completed clip contains only one pig doing the specified behavior.

The video clips in the pig behavior video dataset were extracted from the eighty days’ shooting videos. The dataset included behavioral samples of different pigs from pups to mature. Considering that the length of each raw video saved by the DVR was about 1.5–2 h, which was not suitable for behavior recognition, the videos were cut into clips before used. An example of the process of clipping a 7-second clip is shown in Figure 2. 

We used the video editing software (Apowersoft Video Editor, Apowersoft, Hongkong, China) to clip the whole video into small clips containing different behaviors of a single pig. Specifically, the researchers watched the raw videos (Figure 2a), and determined the starting time of a single pig’s behavior, and clipped the video from the start time. Clips were cut as shown in Figure 2b. After the raw video was cut into clips, the structured background and other pigs’ body should be cut off as much as possible, and the region of interest of the pig involved in the behavior were supposed to be reserved as shown in Figure 2c.

**Figure 3:**
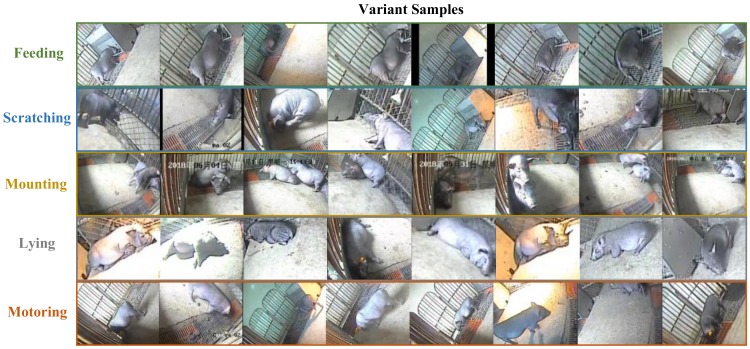
Variant samples of the five behaviors in pig behavior recognition video dataset (PBVD)-5.

Meanwhile, three staff each cut part of the clips for each behavior to minimize subjective factors as much as possible. In order to maintain the diversity of video clips of each behavior, different shooting angles of the same behavior were collected and samples with different perception field sizes were clipped randomly. The samples of the clips are shown in Figure 3. 

### 2.4. Statistics of the PBVD-5

**Figure 4:**
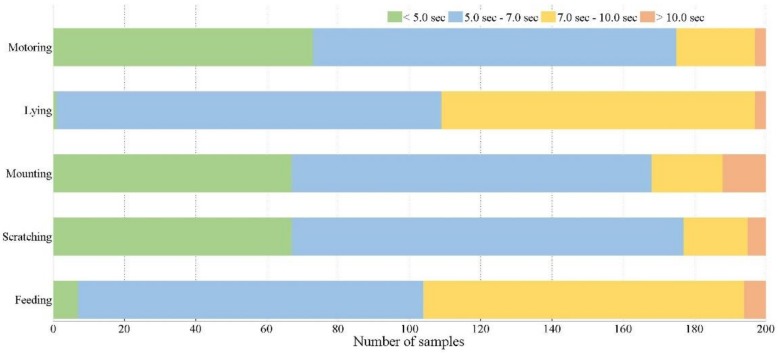
Number of samples per behavior class of pig. The distribution of sample durations is illustrated by the colors.

The PBVD-5 dataset includes five categories of pig behavior, which are feeding, lying, motoring, mounting and scratching, containing 1000 video clips with 200 clips in each category. The resolution of the clips was 320 pixels in length and 240 pixels in width. Each clip contained 25 frames per second, and the video encoder was set to the audio video interleaved format (AVI). The total duration of all the clips was 6276 seconds. The data rate was set to 200 kbps, and the total bit rate was set to 328 kbps. The bar chart of Figure 4 shows the number of clips in each class. The colors on each bar illustrate the durations of different clips included in that class.

As shown in Figure 4, the proportion of time span of various behavior clips in PBVD dataset was also different. Lying behavior is one kind of static behavior, while feeding, motoring, scratching and mounting are four kinds of dynamic behavior. In fact, based on the long-term observation, we found that in addition to the long-term immobility of the pig when lying, the rest of the behavior changed frequently. Therefore, in order to ensure that there was only one kind of behavior in one clip, the clips with dynamic behaviors tended to be shorter in the case of ensuring that the key information of behaviors was not lost. The duration of clips was also related to the size of the perception field. For lying and feeding behaviors, because of the frequent occurrence of multiple pigs piling up and blocking each other, the clips we collected were relatively short.

## 3. Spatiotemporal Convolutional Network for Multi-Behavior Recognition of Pigs

### 3.1. Related Work

Currently, with the continuous development of the research scope of deep learning in computer vision, many research focuses have changed from image classification, object detection and instance segmentation [14,15,16] in two-dimension (2D) images to behavior recognition in spatiotemporal videos. With the proposal of convolutional 3D (C3D), which got simple structure and high efficiency [17], the deformations of spatiotemporal convolution network were widely used in video action recognition of people in standard datasets. Subsequently, on the basis of C3D, the backbone was modified to 3D-Resnet (R3D) by expanding the 2D input and convolutions to 3D [18]. In order to optimize the calculation, a network structure of mixed convolutions (MCx) and reversed mixed convolutions (rMCx) were generated according to R3D by separating a part of 3D convolution layers in different residual stages into two parts. While if each 3D convolution layer was decomposed into a 2D operation in the space dimension and an operation in the temporal dimension, ResNets with the (2 + 1) D convolutions (R (2 + 1) D) network would be generated. According to the experiment results of Tran, the deeper the network was, the better performance R (2 + 1) D provided. However, R3D performed better while the network was shallow [19].

In fact, the information obtained from low frame rate video clips and high frame rate video clips were different. For videos, more effective features could be obtained by extracting features from two pathways with different frame rates. Therefore, Feichtenhofer put forward the SlowFast network, which was divided into two pathways [20]. The SlowFast processed the temporal dimension operation in different residual stages in each pathway with higher temporal resolution and got better results in four standard datasets of human action recognition.

Considering that SlowFast was more appropriate in temporal dimension operation and R3D got better performance in feature extraction. In this paper, the network of SlowFast basic transformation was modified and a multi-behavior recognition network for pigs based on spatiotemporal convolutional network was proposed.

### 3.2. Architecture of the PMB-SCN

On the whole, the architecture of the spatiotemporal convolutional network for pig’s multi-behavior recognition (PMB-SCN) was based on the two pathways structure of SlowFast. On the basis of SlowFast, the backbone was replaced by the two-layer 3D convolution of R3D, which was the basic transformation of SlowFast, and the number of convolution kernel in the residual block was modified appropriately. 

The architecture of the PMB-SCN was mainly composed of three components: two pathways, backbone and the lateral connections.

#### 3.2.1. Pathways of the PMB-SCN

**Figure 5:**
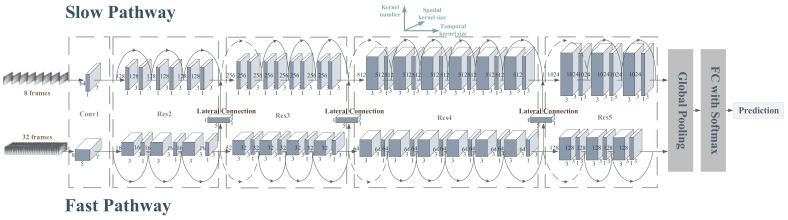
The architecture of spatiotemporal convolutional network for pig’s multi-behavior recognition (PMB-SCN): each cube represented one single convolution operation. The three axes of the cube represented the convolution kernel size of the temporal dimension, the convolution kernel size of the spatial dimension (since the height and the width of the spatial convolution kernel size were the same, it can be represented by one number) and the number of convolution kernels respectively.

Referring to the architecture of SlowFast, the PMB-SCN had two pathways with different temporal speeds, which could be described as a single stream architecture that operates at two different frame rates. The architecture of PMB-SCN is shown in Figure 5.

A The slow pathway of the PMB-SCN

The slow pathway of PMB-SCN had a large temporal stride on input frames, which was 8. This means that it processed only one frame out of 8 frames. The number of frames sampled by the slow pathway was 8. Therefore, the raw clip length was 64 frames. The slow pathway was opted to avoiding temporal downsampling. Simultaneously, the non-degenerate temporal convolutions (temporal kernel size >1) were utilized only in res4 and res5.

B The fast pathway of the PMB-SCN

The fast pathway held the characteristics of high input frame rate and low channel capacity [20]. Specifically, for PMB-SCN, comparing with the slow pathway, the temporal stride on input frames was 2. The number of frames sampled by the fast pathway was 32. Moreover, because of the application of non-degenerate temporal convolutions, the feature tensors had 32 frames along the temporal dimension. Particularly, the fast pathway had 1/8 channels of the slow pathway in PMB-SCN.

#### 3.2.2. Backbone of the PMB-SCN

As is known, the deeper and wider a deep convolutional network is, the better the fitting effect the network reaches. However, this also brings huge consumption of computing resources and over fitting problems. R3D residual network was determined to be the backbone of the PMB-SCN. Table 3 shows the specific network architecture of PMB-SCN.

It can be seen from Table 2 that the backbone of the two pathways of PMB-SCN was of residual network structure with double-layer 3D convolution blocks. This architecture was similar to R3D-34, with the difference that: a) the second layer in each block was actually more like 3D convolution kernels with only 2D operation of spatial feature extraction and b) on the slow pathway, the first layer of the blocks in res2 and res3 stages was 2D convolution operation as well. However, in res4 and res5, the first layer of the blocks was 3D convolution kernels for spatiotemporal feature operation, which was the same as the fast pathway. The convolution kernels of the residual block in each stage were adjusted as well.

#### 3.2.3. Lateral Connections of the PMB-SCN

In order to fuse the features extracted from the slow and the fast pathways, the lateral connection [21] was utilized in this network. In the process of information fusion, the features extracted by the fast pathway were fused into the slow pathway. Specifically, due to the difference between temporal dimension of the fast pathway and the slow pathway, the feature size of the fast pathway was transformed to the same as the slow pathway. The transformation was performed by a 3D convolution, which had a 5 × 1 × 1 kernel with 2 times output channels of the fast pathway, and stride 4 × 1 × 1. Afterwards, the addition fusion was carried out.

Lateral connections were performed after each stage of res2–res4. After the feature extraction of res5, features of each pathway entered into the global average pooling layer, then concatenated before inputted into the fully connection layer. The Softmax function was utilized for classification.

### 3.3. Training and Inference Phase of the PMB-SCN

#### 3.3.1. Training Phase

- Initialization: In the process of PMB-SCN, similar to the SlowFast training process [20], the bias (b) of each layer was initialized as zero. The $y_{l}$ in layer l ($y_{l}=W_{l}x_{l}$, where y was the response at a pixel of the output map, x was a vector of a convolution layer and W represented the weights of a filter and l was to index a layer) was expected to have the same variance for each l. A sufficient condition was:
  (1) $$\frac{1}{2}n_{l}Var\left[w_{l}\right]=1,\forall l$$
  where $n_{l}$ denoted the dimension number of the $x_{l}$. Therefore, the network was initialized by a zero-mean Gaussian distribution, where the standard deviation was $\sqrt{\frac{2}{n_{l}}}$ [22].
- Input data: In terms of temporal domain, the input of the slow pathway and fast pathway were 8 and 32 frames respectively. In terms of the spatial domain, crop with 224 pixels in length and 224 pixels in width was randomly cropped from the videos.
- Training parameters: The batch size was determined to be 8 and the optimizer was synchronized Stochastic Gradient Descent (SGD). The network was trained with batch normalization (BN). The learning rate decay strategy was followed by half-period cosine schedule. The learning rate at the n-th iteration [23] was:
  (2) $$\eta \cdot 0.5\left[\cos\left(\frac{n}{n_{max}}\pi \right)+1\right]$$
  where $n_{max}$ was maximum training iterations and η was base learning rate, which was set to 0.0125. The learning warm-up was also utilized followed SlowFast. The network was trained for 300–400 epochs in variant formation of PMB-SCN and stopped while the accuracy curve of the validation set tended to converge. The momentum was 0.9 and the weight decay was $10^{-4}$ with the dropout was settled as 0.5.

#### 3.3.2. Inference Phase

Following common practice, the data in the test set needed to be clipped before inference phase as the SlowFast network did. In terms of the temporal domain, 10 clips were uniformly sampled from a raw clip along its temporal axis. In spatial domain, crops were scaled by the shorter spatial side, which was 240 pixels and token 3 crops with 240 pixels in length and 240 pixels in width to cover the spatial dimensions. Therefore, each test clip contained 30 views for inference phase.

During inference phase, the parameters of PMB-SCN were initialized by trained model, and only forward process was operated to extract features. The Softmax function was utilized for classification and the average score was used for prediction. The average accuracies of each clip and each sample were calculated for evaluation.

## 4. Materials and Methods of the Experiment

### 4.1. Materials for the Experiment

#### 4.1.1. Experimental Data Acquisition

**Figure 6:**
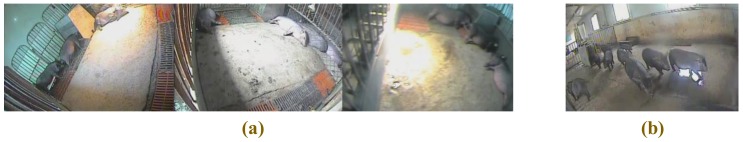
Environment of the three pigsties: (**a**) the pigsties that PBVD-5 was collected from and (**b**) the new pigsty for the experiment.

The experimental data we used came from four pigsties, three of which were used for collecting PBVD-5 and the other was a new pigsty. The environment of the four pigsties is shown in Figure 6. Compared with the pigsties used in PBVD-5, the new pigsty had more pigs and larger site, and its illumination condition and location of the equipment were totally different. The new pigsty could help us analyze the ability of generalization of the networks.

#### 4.1.2. Experimental Dataset Partition

**Figure 7:**
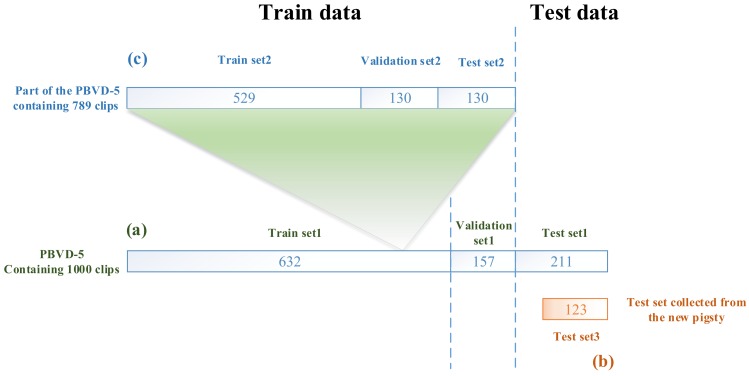
Environmental data partition: (**a**) the PBVD-5 dataset: it was divided randomly into the Train set1, Validation set1 and the Test set1 and used for all networks; (**b**) Test set3: it was collected from the new pigsty and used for all networks and (**c**) part of the PBVD-5 dataset: it consisted of the train set of PBVD-5 and was divided randomly into the Train set2, Validation set2 and Test set2 and used for selecting the optimal architecture of PMB-SCN.

The data utilized in the experiment included 1123 clips, 1000 clips of which were from PBVD-5 and 123 clips from the new pigsty. All the data were used for three experiment purposes, including the selection of the optimal PMB-SCN architecture, the performance test of each network in the same pigsty environment (PBVD-5 test set) and the ability of the generalization test of each trained network in the new pigsty. Figure 7 shows the details of dataset partition.

(a) The partition of the PBVD-5 dataset:

The PBVD-5 containing 1000 clips was randomly divided into a test set containing 211 clips, which was named as Test set1 and a train set with 789 clips. Subsequently, the train set was divided into 4:1 according to stratified sampling and then the Validation set1 with 157 clips was obtained as well as the Train set1 with 632 clips. This partitioned data was used to train all networks and evaluated their performance on PBVD-5.

(b) Test set3:

The Test set3 was collected from the new pigsty, which contained 10 pigs in one pen. The new test set consisted of three categories (limited by different breeding mode and pig sexual maturity, unable to obtain video samples of mounting and feeding behaviors), including 40 scratching samples, 41 motoring samples and 42 lying samples, totaling 123 samples. The Test set3 was utilized to test the networks trained on Train set1 and Validation set1.

(c) Part of the PBVD-5 dataset (partitioned data (c)):

This set of data consisted of the train set of the PBVD with 789 clips and randomly divided into 4:1:1 according to stratified sampling. Then Train set2 with 529 clips, Validation set2 with 130 clips and Test set2 with 130 clips were generated. This partitioned data was used for selecting optimal architecture of PMB-SCN.

The Test set1 and Test set3 used for the final evaluation were strictly ensured that they were not used in the process of training.

#### 4.1.3. Experimental Environment

The hardware platform of this experiment was a small-scale workstation equipped with an Intel Core i9-9900k CPU (Intel, California, USA), a NVIDIA TITAN RTX GPU (NVIDIA, California, USA), 16GB of memory (G.SKILL, Taiwan, China) and a 1 TB solid-state hard disk (SAMSUNG, Gyeonggi, Korea). The operating system for the experiment was Ubuntu 18.04, and used Pytorch1.3 (Facebook, California, USA) with Python3.6.

### 4.2. Experimental Scheme

The experiment of this paper was divided into two parts. The first part contained the experiment of selecting the optimal PMB-SCN on partitioned data (c); In the second part, PMB-SCN was trained on PBVD-5 together with other spatiotemporal convolutional networks and tested on both the Test set1 and Test set3.

#### 4.2.1. Environment Scheme of Selecting the Optimal PMB-SCNs

In order to search for the most suitable architecture of the spatiotemporal convolutional neural network for multi-behavior recognition of pigs, several models were proposed and evaluated. Considering that the depth and width have a vital influence on the performance of deep networks, we conducted the experiments to find the optimal architecture of PMB-SCN. The data used in this experiment was partitioned data (c). The specific architectures involved in this experiment are as follows:

- PMB-SCN-34: basic version of PMB-SCN. The architecture was shown in Section 3.
- PMB-SCN-$34_{half}$: the number of the convolution kernels of the dual pathway in each stage of res2–res5 was changed to half of the original, i.e., the number of the convolution kernels of the slow pathway was set to 64, 128, 256, and 512 for each stage and the fast pathway was set to 8, 16, 32 and 64, while the depth remained 34.
- PMB-SCN-18: the number of the layers was changed to 18. Therefore, the number of residual blocks of each stage from res2–res5 was then 2, 2, 2 and 2 respectively.

#### 4.2.2. Experiment Scheme of Comparing PMB-SCNs with Other State-of-the-Art 3D Networks

The optimal PMB-SCN architecture would be compared with the current advanced single source (RGB) single stream spatiotemporal convolutional networks, which included C3D, MC4 (best performed of MCx according to [19]), rMC4 (best performed of rMCx according to [19]), R (2 + 1) D, R3D and SlowFast-50 on both Test set1 and Test set3 by retraining on the same train set (Train set1 and Validation set1).

The networks are in different layers and used different initialization strategies (pretrained or random initialization). Therefore, for C3D, MC4, rMC4, R (2 + 1) D and R3D, we provided the experimental results with and without pretraining at the same time.

## 5. Experimental Results

### 5.1. Experimental Results for Selecting the Optimal PMB-SCN

**Figure 8:**
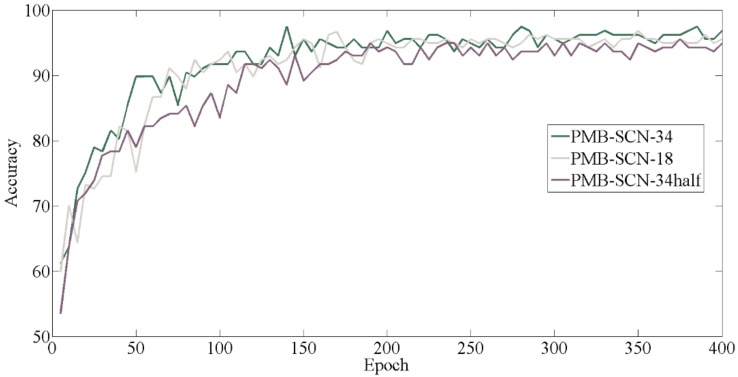
Comparison of the curve of the validation set accuracy.

Three networks with different architectures were trained on Train set2 and Validation set2 to find the optimal structure for PMB-SCN. The curves of the Validation set2 accuracy are shown in Figure 8.

As can be seen in Figure 8, the accuracy of validation set of the three networks converged roughly after 200 epochs. The accuracy curves of the converged validation set showed that the PMB-SCN-34 with more layers and more convolution kernels provided the highest validation accuracy. The network with 18 layers and the same number of convolution kernels (PMB-SCN-18) had a slightly lower accuracy. However, the accuracy of the network with the same number of layer but half number of convolution kernels (PMB-SCN-$34_{half}$) was the lowest. This result was also reflected in the test set metrics, which are shown in Table 4.

It can be seen in Table 4 that reducing either the number of layers or the number of convolution kernels from res2 to res5 stages could decrease the memory occupation and the scale of parameters, but increased the time consumption in result. To be specific, while the network depth was reduced by half, the amount of memory occupation and numbers of parameters would also be reduced by half. If the number of convolution kernels was reduced by half, the memory occupation and parameters would be quartered. This should be attributed to the two pathways of the PMB-SCN. When the number of convolution kernels of the slow pathway was reduced, the parameters of the fast pathway would be reduced accordingly because the ratio of convolution kernels would not change and was constant at eight.

However, the experimental results showed that the reduction of memory occupation and the scale of parameters were at the expense of reducing accuracy and increasing time consumption. Therefore, considering that the main evaluation metrics of each clip were accuracy and test time consumption, PMB-SCN-34 was more suitable than other variants.

### 5.2. Results of the Comparison Between PMB-SCN and Other State-of-the-Art 3D Networks

After finishing the training process of PMB-SCN as well as other state-of-the-art networks on Train set1 and Validation set1. Table 5 shows the specific results of the comparison with the state-of-the-art results for PMB-SCN on Test set1 and Test set3.

#### 5.2.1. Results Analysis on Test Set1 of PBVD

From Table 5, the PMB-SCN provided 3.79% higher Top-1 accuracy and 4% higher views accuracy in comparison to the best performing network without pretrained with single pathway which is R3D-18 on the Test set1 of PBVD. In comparison with the dual pathway network, PMB-SCN provided 0.47% higher Top-1 accuracy and 0.57% higher views accuracy to SlowFast-50. Experimental results show that PMB-SCN and SlowFast with dual pathway could learn more useful features without pretraining.

For single pathway 3D convolutional networks such as MC4, rMC4, R3D and R (2 + 1) D, pretraining on the kinetics dataset was capable of increasing accuracy on the Test set1 of PBVD-5 to a certain extent. Moreover, the networks without pretraining were relatively 3%–4% lower in accuracy. The results show that on PBVD-5, the networks with single pathway were more dependent on pretraining.

**Figure 9:**
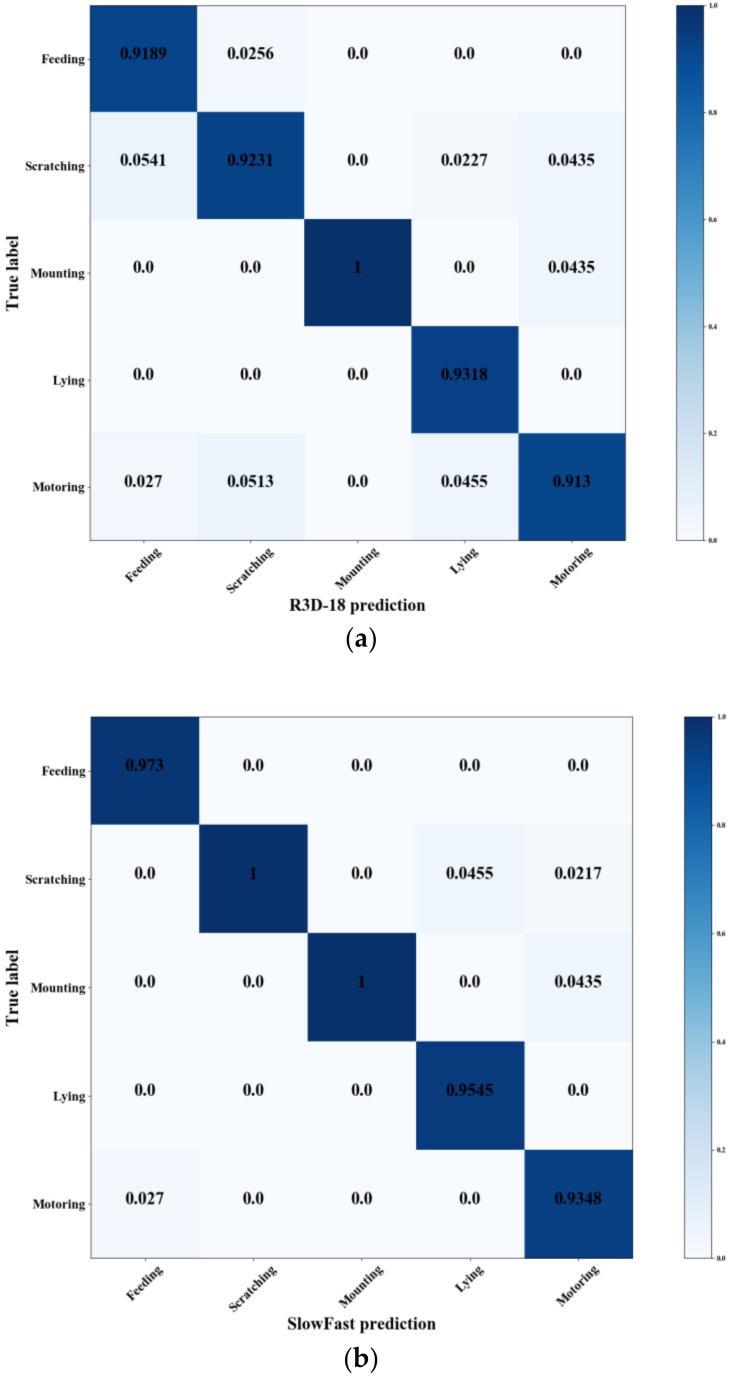

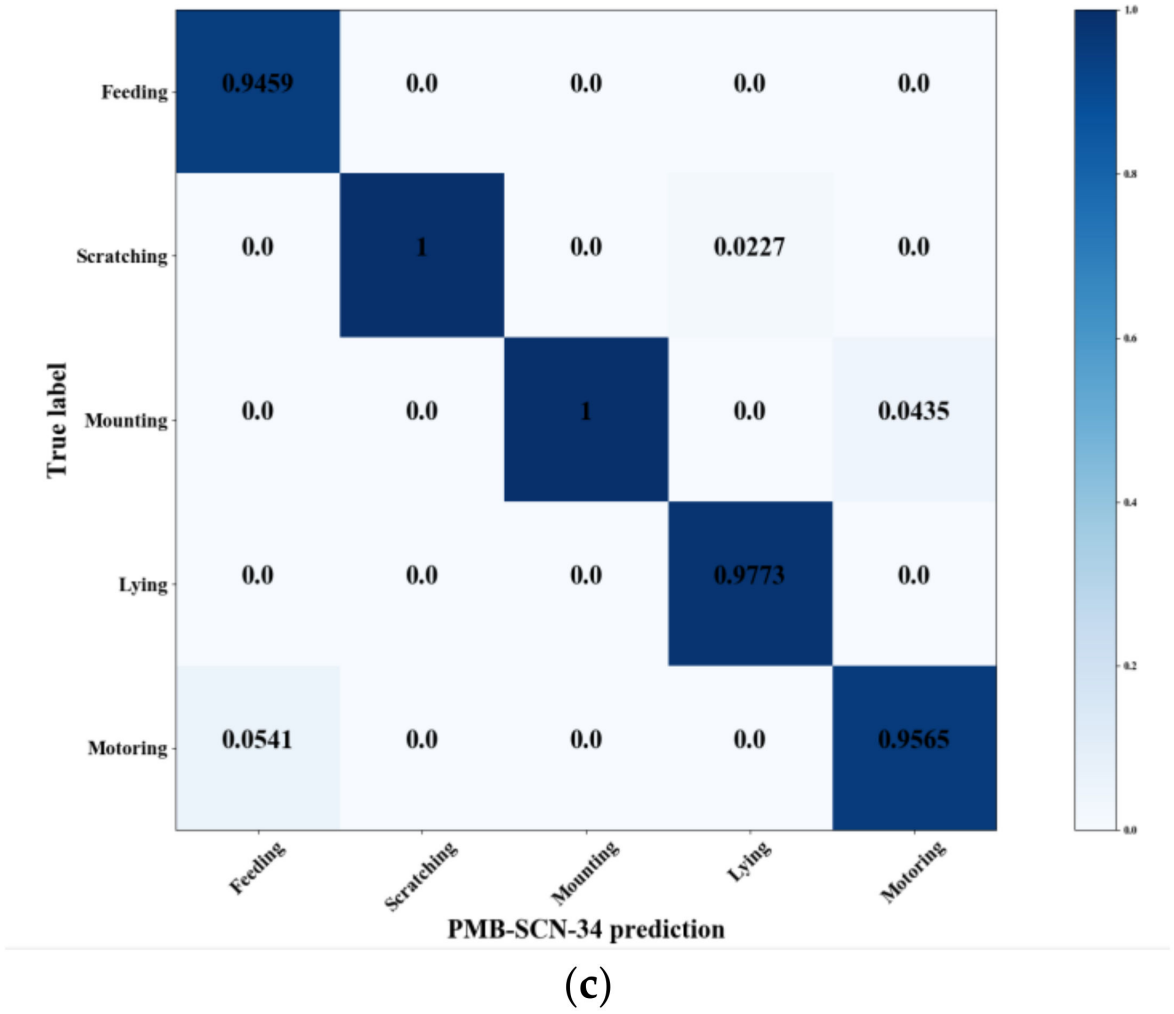
Confusion matrix of the pig behavior classification results of the R3D-18 and the PMB-SCN and the SlowFast on Test set1 of PBVD. (**a**) the confusion matrix of the R3D-18; (**b**) the confusion matrix of the SlowFast; (**c**) the confusion matrix of the PMB-SCN-34.

In order to explore the classification effect of the networks for each behavior, the clips of each category in the Test set1 and the multi-behavior classification results of the R3D-18, SlowFast and PMB-SCN without pretraining were counted. Figure 9 shows the confusion matrix of the different three spatiotemporal convolutional neural networks implemented on Test set1 of PBVD-5.

As shown in Figure 9, it was obvious that in comparison with the R3D-18 network, the classification accuracy of PMB-SCN was equal or higher in each class to some extent. Among them, the classification accuracy of feeding, scratching, lying and motoring behavior was 2.7%, 7.69%, 4.55% and 4.35% higher. Comparing with SlowFast, PMB-SCN provides 2.28% higher in lying behavior recognition and 2.17% higher in motoring behavior recognition.

It could be inferred that in compared with the single pathway networks, networks with dual pathways had the characteristics of high temporal resolution features due to the addition of high frame rate pathway in the temporal domain. We held the opinion that it was helpful in distinguishing pig behaviors with different frequencies. For example, the movement speed of scratching was the fastest but the movement change rule was similar to feeding, while the movement range of scratching was large and was similar to motoring. Compared with other behaviors, the accuracy of the scratching behavior is improved most by the dual pathway networks. Meanwhile, compared with SlowFast, the PMB-SCN improved the recognition accuracy of lying and motoring behaviors. It could be inferred that increasing number of convolutional kernels and convolution layers helps the network to extract more effective spatial features.

**Figure 10:**
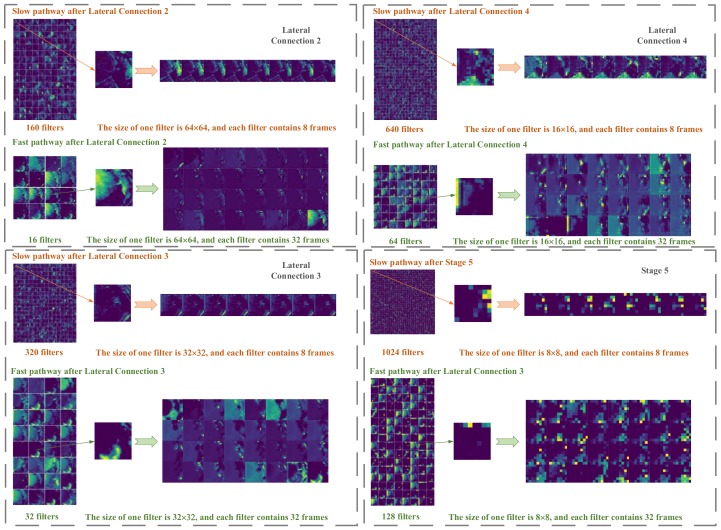
Visualization of the output features of several layers (taking scratching behavior as an example).

Taking scratching behavior as an example, we visualized the output feature of four layers of the PMB-SCN-34, which were Lateral connection 2, 3, 4 and Stage 5 and shown in Figure 10.

#### 5.2.2. Results Analysis on Test Set3

As can be seen from Table 5, PMB-SCN provides 8.94% higher Top-1 accuracy and 10.35% higher view accuracy in comparison to SlowFast-50. Notably, comparing with best performing single pathway network without pretraining, PMB-SCN provided 37.4% higher Top-1 and 31.3% higher view accuracy. The experimental results show that PMB-SCN had a remarkable generalization ability among networks without pretraining.

In comparison to the best performing pretrained single pathway network, which was R (2 + 1) D-34, PMB-SCN shows 0.82% higher Top-1 accuracy but 4.35% lower views accuracy. The experimental results show that comparing the pretrained single pathway networks, the PMB-SCN without pretraining shows equal generalization ability.

It could be inferred that the dual pathway networks indeed provide better generalization ability in pig behavior recognition than networks with single pathway due to the excellent processing ability in temporal domain. In addition, the results show that single pathway networks relied more on pretraining while they were used for a small scale video dataset for behavior recognition.

For further analysis, the specific recognition result of each behavior is counted. The results are shown in Table 6.

As shown in Table 6, PMB-SCN provided 12.19% higher accuracy in motoring behavior recognition, 19.05% higher accuracy in lying behavior but 5% lower accuracy in scratching behavior recognition than SlowFast-50. Due to the fact that the background environment of Train set1 was completely different from that of Test set3, and the PMB-SCN learns more effective features, which might be related to the modification of the number convolution layers and the convolutional kernels that we changed.

It is worth noting that the recognition accuracy of scratching behavior was relatively stable, which was probably due to the fact that scratching behavior had higher frequency than the other two behaviors, and the dual pathway networks had a stronger ability of feature extraction in the temporal domain. However, due to the presence of 10 pigs in one pen, lying behavior might be affected. Therefore, behavior recognition for multiple pigs is still a difficult and promising research direction.

## 6. Discussion

In recent years, deep learning has a rapid development and achieved fruitful results in the field of human action recognition [24,25,26]. Those achievements will not be achieved without the greet contribution of standard datasets such as UCF101 [27], HMDB-51 [28], kinetics [29], etc. to algorithm development and evaluation. A standard dataset will not only be used to evaluate the algorithm but to pretrain the initialization parameters. Therefore, the network could reduce lots of computational power cost and time consumption by using transfer learning.

In the agriculture field, there are also some scholars who have published their dataset on other kind of animals [30], which is undoubtedly a great contribution to the research of animal behavior recognition. Similarly, for the first time, we made the standard dataset of pig behavior videos—PBVD-5. The necessity of making PBVD mainly includes:

a) PBVD contains not only a few routine behaviors, but also a grooming behavior, which is highly concerned in breeding process of pig but has never been researched by previous vision based pig behavior recognition algorithm studies;
b) Videos in PBVD is able to observe the details of the pig’s movements better by using the side-down view while shooting the videos;
c) Since the pig behavior is not complicated as human, PBVD will provide great help for the following scholars in the same field to design and improve the algorithms according to the pig’s motion characteristics, which will not only be a simple transfer learning from human to animal.

In fact, this is the first time that a pig behavior recognition video dataset has been proposed, so in the process of making PBVD, we referred to the standard dataset of UCF101 [27] to a large extent including many parameters such as the length of clips, resolution and video format. We were cutting longer clips and clips captured by infrared at night to complement it. 

In view of the outstanding contributions of deep learning in the field of vision, a large number of scholars hope to transplant the algorithms of deep learning into animal behavior recognition. However, most of the current researches are to obtain the top view images, aiming at the detection of the region of interests of the 2D spatial images. These methods rely too much on the inner environment of the pig pen, and has strong dependence on the structured environment. The simple algorithm transplantation will not achieve considerable results while the pen is replaced. In fact, the judgments of disease also require some auxiliary behavior monitoring, such as grooming behavior. However, region-based behavior monitoring is unable to achieve this goal. These problems make the research of spatiotemporal convolutional neural network have vital significance:

a) 3D convolutional neural network is a kind of behavior recognition method that focuses on the characteristics of the behavior itself. Even if the position of pig remains unchanged, the behavior like scratching can still be detected. This behavior monitoring method is more in line with the nature of behavior recognition.
b) The input of 3D convolutional network is video clips, and the temporal domain operation is added in the process of extracting features. The feature extraction operation in the temporal domain can extract the sequential features of one behavior in temporal axis, which is impossible for 2D images.
c) When this 3D convolutional network is used together with object detection, pig detection and pig behavior recognition can be performed simultaneously in the same video. This will be able to provide us with some daily behavior statistical functions. Additionally, the time consumption of 3D convolutional network is also considerably small, thus it will be able to meet the needs of follow-up real-time monitoring.

In general, the 3D convolutional neural network for pig behavior recognition proposed in this paper can not only make an important contribution to the follow-up long video analysis, but also provide a basis for real-time behavior recognition based on action characteristics simultaneously implementing pig detection.

## 7. Conclusions

In this paper, a standard dataset of pig’s behavior videos, which is called PBVD-5, was built containing five categories of behaviors including feeding, lying, motoring, mounting and scratching. 

A spatiotemporal convolutional neural network called PMB-SCN was proposed based on the basic transformation of the SlowFast for multi-behavior recognition of pigs. Variants of PMB-SCN were implemented and the optimal architecture reached a top-1 accuracy of 97.63%, a views accuracy of 96.35%, which shows that this algorithm provides the possibility for the follow-up real-time monitoring.

Six kinds of the state-of-the-art 3D convolutional neural networks, including those with and without pretraining, were trained on PBVD-5 and compared with PMB-SCN on a new test set collected from a completely different pigsty. Experimental results show that PMB-SCN provided a top-1 accuracy of 91.87% and a view accuracy of 84.47%, which indicates that this network had a certain ability of generalization and do learn the essence of the pig’s behavior.

In the end, we would like to emphasize that the dataset of PBVD-5 and the proposed spatiotemporal convolutional neural network of PMB-SCN in this paper has laid a solid foundation for the follow-up statistical analysis of long video behavior and real-time behavior recognition simultaneously implementing pig detection.

## Figures and Tables

**Table 1:** Specific information of pigs.

Camera Number	Pig Identifier	Date of Birth	Age (Days)	Weight (kg)
a	1	25 October 2017	150	9.75
	2	26 November 2017	118	12.5
	3	19 December 2017	95	13.15
b	4	9 November 2017	135	13.1
	5	13 December 2017	101	15.85
	6	15 December 2017	99	13.7
c	7	4 November 2017	140	16.09
	8	2 December 2017	112	10.65
	9	2 December 2017	112	16.39

Age is the number of live days of the pigs by 23 March 2018. Weight is the initial weight measured on 24 March 2018.

**Table 2:** Definition and description of pig behaviors.

Activity Level	Behavior	Description
Moving	Feeding	Head into the feeding trough and swing up and down. [11]
	Motoring	Walking, running or jumping. [12]
	Mounting	One pig lifts its two front legs and puts these or its sternum on any part of the body or head of another pig, is the most widely used indicator of reproductive behavior for estrus detection. [13]
	Scratching	Rubbing body against pen fixtures or rolling on the ground. [12]
Staying	Lying	Head resting against the ground and not moving, body (parts) may make sharp, sudden, short-lasting movements. [12]

**Table 3:** The specific architecture of the PMB-SCN based on SlowFast.

Stage	Output Sizes	Slow Pathway	Fast Pathway
raw clip	64 × 24 × 224	-	
data layer	Slow: 8 × 224 × 224	stride 8 × 1 × 1	stride 2 × 1 × 1
	Fast: 32 × 224 × 224		
conv1	Slow: 8 × 112 × 112	1 × 7 × 7, 64	5 × 7 × 7, 8
	Fast: 32 × 112 × 112	stride 1 × 2 × 2	stride 1 × 2 × 2
pool1	Slow: 8 × 56 × 56	1 × 3 × 3, max	
	Fast: 32 × 56 ×56	stride, 1 × 2 × 2	
res2	Slow: 8 × 56 × 56	$\left[\begin{array}{c} 1\times 3\times 3,\;128\\ 1\times 3\times 3,\;128 \end{array}\right]\times 3$	$\left[\begin{array}{c} 3\times 3\times 3,\;16\\ 1\times 3\times 3,\;16 \end{array}\right]\times 3$
	Fast: 32 × 56 × 56		
res3	Slow: 8 × 28 × 28	$\left[\begin{array}{c} 1\times 3\times 3,\;256\\ 1\times 3\times 3,\;256 \end{array}\right]\times 4$	$\left[\begin{array}{c} 3\times 3\times 3,\;32\\ 1\times 3\times 3,\;32 \end{array}\right]\times 4$
	Fast: 32 × 28 × 28		
res4	Slow: 8 × 14 × 14	$\left[\begin{array}{c} 3\times 3\times 3,\;512\\ 1\times 3\times 3,\;512 \end{array}\right]\times 6$	$\left[\begin{array}{c} 3\times 3\times 3,\;64\\ 1\times 3\times 3,\;64 \end{array}\right]\times 6$
	Fast: 32 × 14 × 14		
res5	Slow: 8 × 7 × 7	$\left[\begin{array}{c} 3\times 3\times 3,\;1024\\ 1\times 3\times 3,\;1024 \end{array}\right]\times 3$	$\left[\begin{array}{c} 3\times 3\times 3,\;128\\ 1\times 3\times 3,\;128 \end{array}\right]\times 3$
	Fast: 32 × 7 × 7		
	1 × 1 × 1	Spatiotemporal pooling, concatenate fc layer with softmax	

**Table 4:** Performance of the experiments of selecting optimal PMB-SCN on the Test set2.

Model	Kernel Number of the Slow Pathway	Layer	Top-1 Accuracy (%)	Views Accuracy (%)	Memory (Mb)	Parameters (Mb)	Time Consumption (ms)
PMB-SCN	[64,64,128,256,512]	34	96.92	96.10	160.19	41.41	95.77
	[64,128,256,512,1024]	34	98.46	96.59	637.87	165.46	50.11
	[64,128,256,512,1024]	18	97.69	97.26	330.14	86.03	88.55

The top-1 accuracy indicated the percentage of the same number of softmax predictions as the labels in the test set. The views accuracy represented the percentage of correct prediction in the total views.

**Table 5:** Comparison of the performance of different state-of-the-art 3D networks on the test sets.

Model	Pretrain	Test Set3		Test Set1	
		Top-1 Accuracy (%)	Views Accuracy (%)	Top-1 Accuracy (%)	Views Accuracy (%)
C3D-16	Kinetics	86.18	82.28	95.26	94.00
MC4-18	Kinetics	86.99	85.00	96.68	96.11
rMC4-18	Kinetics	73.17	74.27	96.21	95.88
R(2+1)D-18	Kinetics	76.42	76.42	97.16	96.35
R(2+1)D-34	Kinetics	91.05	88.82	96.68	95.76
R3D-18	Kinetics	86.18	85.08	97.16	96.82
MC4-18	-	54.47	53.17	92.89	91.94
rMC4-18	-	35.77	37.15	92.42	91.70
R(2+1)D-18	-	50.41	46.91	93.36	92.06
R3D-18	-	53.66	52.97	93.84	92.35
R3D-34	-	43.90	41.10	93.36	91.99
SlowFast 8×8, R50	-	82.93	74.12	97.16	95.78
PMB-SCN	-	91.87	84.47	97.63	96.35

The first column showed the name of the model participating in comparison test, and the second column showed the name of the standard dataset from which the pretrained model was used. The top-1 accuracy indicated the percentage of the same number of Softmax predictions as the labels in the test set. The views accuracy represented the percentage of correct prediction in the total views.

**Table 6:** Results of the new dataset.

Ground Truth		Predicted Class							
		Motoring	Scratching	Lying	Mounting	Feeding	Precision Rate (%)	Top-1 Accuracy (%)	Views Accuracy (%)
SlowFast	Motoring	34	1		6		82.93	82.93	74.12
	Scratching	1	38		1		95		
	Lying	3	3	30	4	2	71.43		
PMB-SCN	Motoring	39			2		95.12	91.87	84.47
	Scratching	1	36	2	1		90		
	Lying	4		38			90.48		

The top-1 accuracy indicated the percentage of the same number of Softmax predictions as the labels in the test set. The views accuracy represented the percentage of the correct prediction in the total views.
